# Multiple coronary thrombosis

**DOI:** 10.15171/jcvtr.2016.19

**Published:** 2016-06-30

**Authors:** Mohammad Ali Ostovan, Pooyan Dehghani

**Affiliations:** ^1^Department of Cardiology, School of Medicine, Shiraz University of Medical Sciences, Shiraz, Iran; ^2^Cardiovascular Research Center, Shiraz University of Medical Sciences, Shiraz, Iran

We read the article “*Simultaneous thrombosis of multiple coronary arteries in a patient with rheumatoid arthritis*” by Kalayci et al^1^ with great interest. The authors presented a patient with rheumatoid arthritis treated by methotrexate developing ST segment elevation myocardial infarction. We commend the authors for this interesting report and want to make some comments.

Between the three options authors had at time of performing coronary angiography, it was the wisest choice to administer fibrinolytic therapy; but other causes of simultaneous thrombosis in coronary arteries should be ruled out. It might have been better that the authors had performed a transesopheageal echocardiography (TEE) to rule out the presence of left atrial (LA) or left ventricular (LV) clot and also probable paradoxical embolism due to patent foramen ovale.^2^ In the presence of an undiagnosed large LA/LV thrombosis, a catastrophe could have happened.

Occlusion of wrap around left anterior descending artery at its midpart, by itself, could justify the presence of ST segment elevations in anterior and inferior leads. So the presence of thrombosis in the left main and right coronary arteries could have another story. Although very rare, in situ thrombosis formation in the catheter or on the guidewire and embolization to coronaries with injection^3^ might be the cause of left main or RCA clots though not justifying all thromboses in this case. Careful examination of catheters and guidewires should have been done at the time.

Many other predisposing factors for multiple coronary thromboses have been suggested, such as cocaine or amphetamine use, high serum levels of cathecholamines, essential thrombocytosis and hyperhomocysteinemia,^4^ that should have been evaluated in this case.

As the authors nicely mentioned, there are just few reports concerning arterial thrombosis formation with methotrexate therapy but it reduces overall cardiovascular disease burden in patients with rheumatoid arthritis. Another drug that could be responsible for this situation in patients with RA is corticosteroid not mentioned in the case.

In conclusion, we think that the authors managed the patient well but performing TEE before administration of fibrinolytic therapy was mandatory. Meanwhile, according to the current guidelines,^5^ we recommend repeating coronary angiogram in less than 24 hours as a pharmacoinvasive approach, in order to address the probable remained stenosis or occlusion earlier.

## Ethical issues

Not applicable.

## Competing interests

Authors declare no conflict of interest in this study.
